# An Elimination Method of Temperature-Induced Linear Birefringence in a Stray Current Sensor

**DOI:** 10.3390/s17030551

**Published:** 2017-03-09

**Authors:** Shaoyi Xu, Wei Li, Fangfang Xing, Yuqiao Wang, Ruilin Wang, Xianghui Wang

**Affiliations:** 1 School of Mechanical and Electrical Engineering, China University of Mining and Technology, Xuzhou 221116, China; xutianyia@126.com (S.X.); shi_xian_zhong@163.com (Y.W.); wangruilin93@hotmail.com (R.W.); cumtwxh@hotmail.com (X.W.); 2 School of Information and Electrical Engineering, China University of Mining and Technology, Xuzhou 221116, China; 3 School of Mechatronic Engineering, Xuzhou College of Industrial Technology, Xuzhou 221116, China; cumtxff@cumt.edu.cn

**Keywords:** stray current sensor, temperature-induced linear birefringence, geometric rotation effect

## Abstract

In this work, an elimination method of the temperature-induced linear birefringence (TILB) in a stray current sensor is proposed using the cylindrical spiral fiber (CSF), which produces a large amount of circular birefringence to eliminate the TILB based on geometric rotation effect. First, the differential equations that indicate the polarization evolution of the CSF element are derived, and the output error model is built based on the Jones matrix calculus. Then, an accurate search method is proposed to obtain the key parameters of the CSF, including the length of the cylindrical silica rod and the number of the curve spirals. The optimized results are 302 mm and 11, respectively. Moreover, an effective factor is proposed to analyze the elimination of the TILB, which should be greater than 7.42 to achieve the output error requirement that is not greater than 0.5%. Finally, temperature experiments are conducted to verify the feasibility of the elimination method. The results indicate that the output error caused by the TILB can be controlled less than 0.43% based on this elimination method within the range from −20 °C to 40 °C.

## 1. Introduction

**Figure 1:**
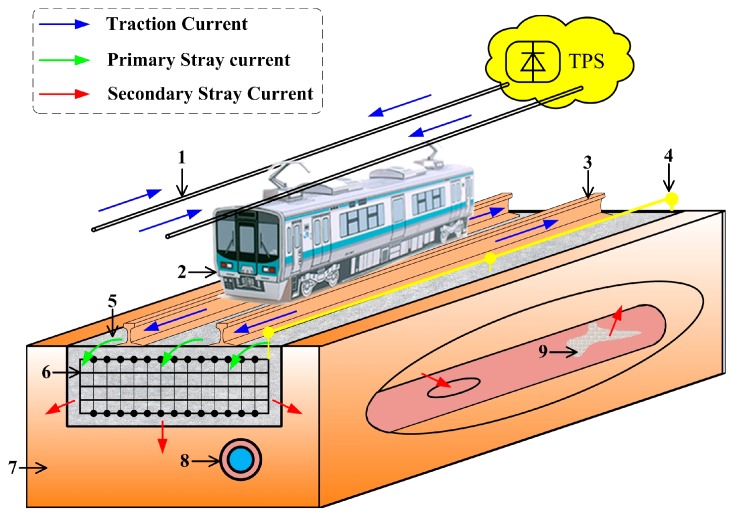
Schematic diagram of stray current formation mechanism: 1. Overhead lines; 2. Train; 3. Running rail; 4. Cable for returning the primary stray current; 5. Track bed; 6. Stray current control mat; 7. Soil; 8. Buried pipe; 9. Corrosion part of buried pipe.

Nowadays, urban rail transit systems are vastly utilized in modern cities of the world due to their high capability and capacity in transferring passengers, air pollution reduction, traffic reduction in crowded cities, optimal utilization of energy resources, and their fast speed [1]. For the purpose of reducing the construction cost and the maintenance cost, running rails usually act as the return path of traction current in the urban rail transit system. The running rails have limited conductivity and are not completely insulated over the ground, so a part of the traction current will flow into the earth without returning to the traction substation (TPS). This kind of current underground is called ‘stray current’. The stray current can be divided into two parts. The current leaving running rails will flow into the stray current control mat, which is called the primary stray current. Most of the primary stray current will return to the TPS again, the remaining current will flow into the soil beneath, which is called the secondary stray current [2]. If the secondary stray current flows into the buried pipe, the corrosion of buried pipe will occur from each point that current transfers from the buried pipe to the electrolyte [3]. The stray current formation mechanism is shown in Figure 1. It is noted that there have been a lot of severe cases concerning secondary stray current corrosion. For example, after starting the operation of the Guangzhou metro line 1 in 1999, the maintenance of the mid-pressure gas network built by Guangzhou Gas Group Co. Ltd. has gone up substantially. The maintenance cases numbered about 13 in 1998 while they were about 248 in 2011, which have been proven to be closely related to the secondary stray current corrosion from the Guangzhou metro line 1. According to our test results in Guangzhou metro at a constant temperature (about 20 °C), the maximum value of the secondary stray current is about 28.94 A while its minimum value is about 14.07 A. Thus, there is a secondary stray current measurement requirement to minimize its impact on the buried pipe.

The optical fiber current sensor (OFCS) has many advantages compared with the traditional electric sensors, including inherent safety, passive type, and anti-corrosion. It has attracted wide attention in the past three decades. For example, an OFCS has been proposed based on the Fabry-Perot interferometer using a fiber Bragg grating demodulation [4]; an OFCS has been reported based on a long-period fiber grating with a permanent magnet [5]; and an OFCS has been designed based on microfiber and chrome-nickel wire [6]. Moreover, the most widely used OFCS mechanism by far, the Faraday effect, which has been explored in many configurations [7,8,9,10]. In this case, the light propagating in a sensing fiber experiences a rotation in the angle of polarization in the presence of an external magnetic field. According to the Faraday effect, we have proposed the stray current sensor [11,12], which has been successfully applied for the secondary stray current measurement in Guangzhou metro. The main problem in the practical application is that the measurement accuracy of the stray current sensor is greatly limited by the temperature-induced linear birefringence (TILB). As far as we know, there are four effective methods that have improved elimination of the TILB. The first method applies the thermally annealed fiber as the sensing fiber [8]. However, the thermally annealed fiber is found to be easily damaged. The second method applies the twisted fiber as the sensing fiber [13]. However, the effect of this method is gradually decreased over time due to the release of the shearing stress produced by the torsion operation. The third method applies highly spun birefringence fiber as the sensing fiber [14]. However, the Verdet constant of this fiber is difficult to be evenly distributed. Moreover, this fiber needs to be improved in some ways, such as the high polarization mode dispersion and the high cost of fabrication. The last method applies the Faraday mirror to eliminate the TILB. However, this method is effective only when the Faraday rotation angle (single pass) is 45 deg [15]. In fact, this Faraday rotation angle is very sensitive to the external magnetic field [16].

In this paper, an elimination method of the TILB in the stray current sensor is proposed. This method follows the idea that the sensing fiber in the sensor head is designed as the CSF based on geometric rotation effect, which produces a large amount of circular birefringence to suppress the TILB. The structure of this paper is arranged as follows: Firstly, the configuration and principle of the stray current sensor are demonstrated. Then, the differential equations that indicate the polarization evolution of the CSF element are derived. The output error model is built based on the Jones matrix calculus. On these bases, an accurate search method is proposed to obtain the key parameters of the CSF, including the length of the cylindrical silica rod and the number of the curve spirals. Finally, temperature experiments are conducted to verify the feasibility of this elimination method.

## 2. Configuration and Principle of Stray Current Sensor

**Figure 2:**
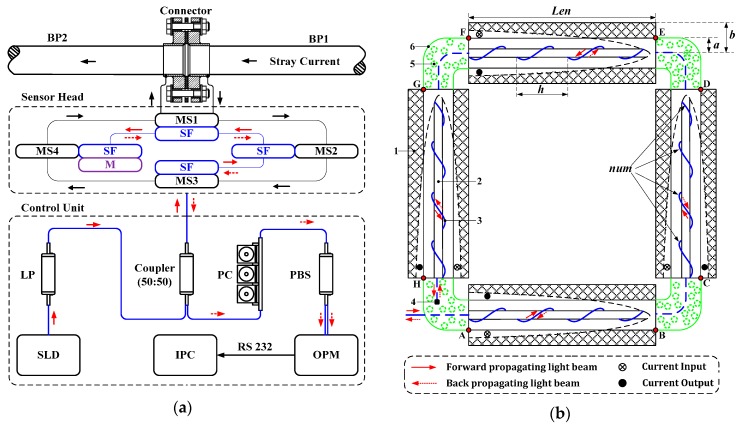
Configuration of the stray current sensor and its sensor head: (**a**) Configuration of stray current sensor: SF represents the CSF; M represents the mirror; MS1, MS2, MS3, and MS4 represent four multilayer solenoids in series; (**b**) Configuration of the sensor head: 1. Multilayer solenoid; 2. Cylindrical silica rod; 3. CSF; 4. Mirror; 5. Heat-insulated cotton; 6. Plastic bend.

The configuration of stray current sensor is schematically illustrated in Figure 2a. The light wave from the super luminescent diode (SLD) first enters into the polarizer (LP) to produce the linearly polarized light. The linearly polarized light is then coupled into the sensor head. The sensor head is composed of the multilayer solenoids, the sensing fiber, and a mirror. The mirror is attached at the end of the sensing fiber. When the stray current is applied on the solenoids, the magnetic field is induced. With the help of the reflection in the mirror, two Faraday effects occur in the sensor head. After that, carrying the Faraday rotation information, the reflected polarized light is coupled into the polarization beam splitter (PBS) to be split into the orthogonally polarized optical signal, which is detected by the optical power meter (OPM). These detection results are sent to the industrial personal computer (IPC) through RS232 interface. It is noted that the polarization controller (PC) is set between the coupler and the PBS to modulate the plane of polarization through 45 degrees. Moreover, a connector with an insulation washer is installed between buried pipeline 1 (BP1) and BP2. It causes the stray current in BP1 to flow into the sensor head and then reach BP2.

The detailed configuration of the sensor head is shown in Figure 2b. There are four multilayer solenoids installed in the sections AB, CD, EF, and GH, respectively. It is noted that the solenoids are connected in series and the induced magnetic field in each solenoid is considered to be equivalent. The sensing fiber in each section is designed to wind along the non-planar curve on a cylindrical silica rod, which is used to produce a large amount of circular birefringence to suppress the effect of TILB. Moreover, there are four plastic bends installed in sections BC, DE, FG, and HA, respectively. Each bend is filled with heat-insulated cotton. The sensing fiber is freely placed in each bend.

## 3. Output Error Mode of Stray Current Sensor

In our sensor, the circular birefringence is produced based on the geometric rotation effect [17,18]. This effect is described in that its polarization orientation will rotate and the rotation angle is equal to the integration of the torsion along the curve when the linearly polarized light propagates along the sensing fiber shaping into the non-planar curve. In the sections AB, CD, EF, and GH of the sensor head shown in Figure 2b, the non-planar curves are all the cylindrical spiral curves. The sensing fiber is called the cylindrical spiral fiber (CSF) in this work. The parameter equation of the CSF can be given by

(1) $$f\left(\varepsilon \right)=f_{x}\cdot i+f_{y}\cdot j+f_{z}\cdot k=r\cos\left(num\cdot \varepsilon \right)\cdot i+r\sin\left(num\cdot \varepsilon \right)\cdot j+\frac{h\cdot num\cdot \varepsilon }{2\pi }\cdot k,$$

where *ε* is the angle that makes a full circle, 0 ≤ *ε* ≤ 2π; *r* is the radius of the cylindrical silica rod, mm; *num* is the number of the curve spirals; *h* is the length of each curve spiral, mm; *f_x_* is equal to *r*cos(*num*·*ε*); *f_y_* is equal to *r*sin(*num*·*ε*); and *f_z_* is equal to (*h*·*num*·*ε*)/(2π).

The torsion *τ* and the curvature *μ* of the CSF can be derived by

(2) $$\tau =\frac{\left(f^{\prime },f^{\prime \prime },f^{\prime \prime \prime }\right)}{{\left(f^{\prime }\times f^{\prime \prime }\right)}^{2}}=\frac{2\pi h}{h^{2}+4\pi^{2}r^{2}},\;\mu =\frac{\left|f^{\prime }\times f^{\prime \prime }\right|}{{\left|f^{\prime }\right|}^{3}}=\frac{4\pi^{2}r}{h^{2}+4\pi^{2}r^{2}},$$

where *f’*, *f’’* and *f’’’* represent the first derivative, the second derivative, and the third derivative, respectively; and (*f’*, *f’’*, *f’’’*) represents the mixed product of *f’*, *f’’*, and *f’’’*.

Moreover, the arc length of the infinitesimal vector **d***l* at *ε* can be given by:

(3) $$dl=\sqrt{{\left(f_{x}^{\prime }\right)}^{2}+{\left(f_{y}^{\prime }\right)}^{2}+{\left(f_{z}^{\prime }\right)}^{2}}\cdot d\varepsilon =\rho \cdot d\varepsilon =\frac{num}{2\pi }\cdot \sqrt{4\pi^{2}r^{2}+h^{2}}\cdot d\varepsilon ,$$

The magnetic field intensity on **d***l* can be simplified by [12]

(4) $$H=n_{1}\cdot n_{2}\cdot c\cdot \ln\left[\left(b+\sqrt{b^{2}+c^{2}}\right)/\left(a+\sqrt{a^{2}+c^{2}}\right)\right]\cdot Cur,$$

where *Cur* is the stray current applied on the solenoid; *c* is defined as *Len*/2; and *n*_1_ and *n*_2_ are equal to *N*_1_/*Len* and *N*_2_/(*b*–*a*), respectively. Among them, *a* and *b* represent the internal and external diameter of the solenoid, respectively. *Len* represents the length of the cylindrical silica rod. *N*_1_ and *N*_2_ represent the number of turns of solenoid along the axial and vertical direction.

Since the CSF element **d***l* is infinitesimal, the linear birefringence, circular birefringence and Faraday rotation angle are distributed evenly in **d***l*. Thus, for the forward propagating light beam, the Jones matrix of the CSF element can be obtained as

(5) $$J_{f}\approx \left[\begin{array}{cc} 1+jq\rho \cdot d\varepsilon & -\left(\tau \rho +VH\rho \right)\cdot d\varepsilon \\ \left(\tau \rho +VH\rho \right)\cdot d\varepsilon & 1-jq\rho \cdot d\varepsilon \end{array}\right],$$

where *q* is equal to LB/2; LB represents the linear birefringence per unit length, rad/m; *V* is the Verdet constant of the sensing fiber, which is about 0.73 μrad/A at 1550 nm [7]. Since the CSF is wound on a cylindrical silica rod, the LB includes the bending-induced linear birefringence (BILB). Because the bending radius can be measured, the BILB per unit length is usually obtained as [19]

(6) $$\mathrm{BILB}=\frac{\pi n^{3}}{2\lambda }\left(p_{12}-p_{11}\right)\left(1+\nu \right)\kappa^{2}\cdot \mu^{2},$$

where, *λ* is the wavelength of the input light, *λ* = 1.55 µm; *n* is the refractive index of the fiber core, *n* = 1.458; *p*_11_, _12_ are the strain-optical coefficients, *p*_11_ = 0.121 and *p*_12_ = 0.270; *ν* is the Poisson’s ratio, *ν* = 0.17; *κ* is the equivalent radius of the sensing fiber, *κ* = 62.5 µm; *μ* is the curvature of the CSF that can be calculated based on Equation (2).

It is known that the other component of the linear birefringence LB is the TILB when the stray current sensor works in varying temperature conditions. The TILB per unit length can be obtained as [20]

(7) $$\mathrm{TILB}=\frac{\pi n^{3}}{\lambda }\left(p_{12}-p_{11}\right)\left(1+\nu \right)\cdot \frac{\Delta \sigma }{E_{1}},$$

where, *E*_1_ is the Young’s modulus of the CSF, *E*_1_ = 7.0 × 10^10^ N/m^2^; ∆*σ* is the stress difference between the orthogonal principal axes of the CSF, which is obtained as

(8) $$\Delta \sigma =E_{2}\cdot \eta \cdot \left(T-T_{0}\right),$$

where, *E*_2_ is the Young’s modulus of the cylindrical silica rod, *E*_2_ = *E*_1_ in this work; *η* is the linear expansion coefficient of the cylindrical silica rod, *η* = 5.0 × 10^−7^ /K; *T* is the work temperature of the stray current sensor, which is within a range from −20 °C to 40 °C; and *T*_0_ is the room temperature, *T*_0_ = 20 °C. Thus, the LB of the CSF element is equal to (TILB + BILB)d*l*.

For the back propagating light beam, the Jones matrix of the CSF element can be obtained as

(9) $$J_{b}\approx \left[\begin{array}{cc} 1-jq\rho \cdot d\varepsilon & \left(\tau \rho -VH\rho \right)\cdot d\varepsilon \\ -\left(\tau \rho -VH\rho \right)\cdot d\varepsilon & 1+jq\rho \cdot d\varepsilon \end{array}\right],$$

**Figure 3:**
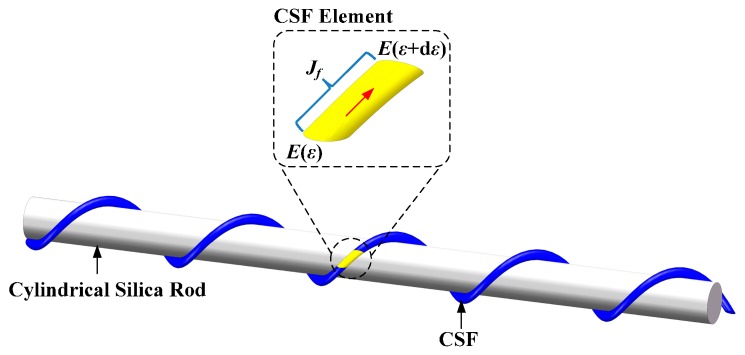
Polarization evolution of the CSF element.

The polarization evolution of the CSF element is shown in Figure 3. For the forward propagating light, the complex amplitude of the input vector is assumed as *E*(*ε*) = [*E_x_*(*ε*); *E_y_*(*ε*)] while the complex amplitude of the output vector is assumed as *E*(*ε* + d*ε*) = [*E_x_*(*ε* + d*ε*); *E_y_*(*ε* + d*ε*)]. According to the Jones calculus and Equation (5), the differential equations can be obtained as

(10) $$\left\{ \begin{array}{l} E_{x}^{\prime }\left(\varepsilon \right)=jq\rho \cdot E_{x}\left(\varepsilon \right)-\left(\tau \rho +VH\rho \right)\cdot E_{y}\left(\varepsilon \right)\\ E_{y}^{\prime }\left(\varepsilon \right)=\left(\tau \rho +VH\rho \right)\cdot E_{x}\left(\varepsilon \right)-jq\rho \cdot E_{y}\left(\varepsilon \right) \end{array}\right.,$$

A similar approach can be taken for the back propagating light. The complex amplitude of the input vector is assumed as *A*(*ε*) = [*A_x_*(*ε*); *A_y_*(*ε*)] while that of the output vector is assumed as *A*(*ε* + d*ε*) = [*A_x_*(*ε* + d*ε*); *A_y_*(*ε* + d*ε*)]. The differential equations can be obtained as

(11) $$\left\{ \begin{array}{l} A_{x}^{\prime }\left(\varepsilon \right)=-jq\rho \cdot A_{x}\left(\varepsilon \right)+\left(\tau \rho -VH\rho \right)\cdot A_{y}\left(\varepsilon \right)\\ A_{y}^{\prime }\left(\varepsilon \right)=-\left(\tau \rho -VH\rho \right)\cdot A_{x}\left(\varepsilon \right)+jq\rho \cdot A_{y}\left(\varepsilon \right) \end{array}\right.,$$

**Figure 4:**
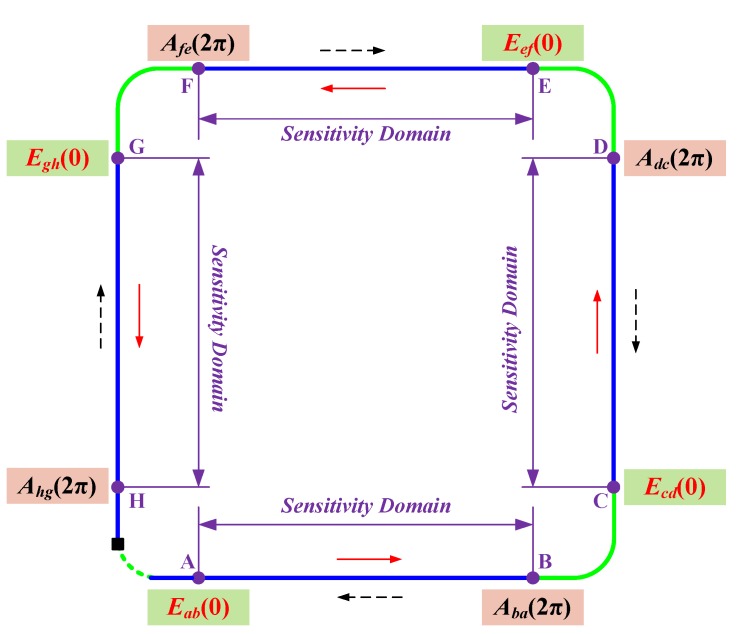
The initial condition of the differential equation of the CSF in each section: (1) for the forward propagating light, *E*_ab_(0), *E*_cd_(0), *E*_ef_(0), and *E*_gh_(0) are the initial conditions in sections AB, CD, EF, and GH; (2) for the back propagating light, *A*_ab_(2π), *A*_cd_(2π), *A*_ef_(2π), and *A*_gh_(2π) are the initial conditions in sections AB, CD, EF, and GH.

Figure 4 shows the initial condition of the differential equation of the CSF in each section. For the forward propagating light beam in section AB, the complex amplitude of the input vector is known at point A (*ε* = 0), which is defined as *E*_ab_(0). The differential equation shown in Equation (10) is calculated based on this known condition. According to the calculation result, the complex amplitude of the output vector can be obtained at point B (*ε* = 2π), which is defined as *E*_ab_(2π). Since the sensing fiber in section BC is short and freely placed, the polarization of light is considered to be unchanged. Thus, in section CD, the complex amplitude of the input vector at point C is the same as *E*_ab_(2π), which is defined as *E*_cd_(0). Referring to the calculation method of *E*_ab_(2π), the complex amplitude of the output vector can be obtained at point D, which is defined as *E*_cd_(2π). Similarly, *E*_ef_(0), *E*_ef_(2π), *E*_gh_(0), and *E*_gh_(2π) are obtained. Moreover, for the back propagating light beam, *A*_hg_(2π), *A*_hg_(0), *A*_fe_(2π), *A*_fe_(0), *A*_dc_(2π), *A*_dc_(0), *A*_ba_(2π) and *A*_ba_(0) are obtained in turn based on Equation (11). It is noted that *A*_hg_(2π) is the product of *E*_gh_(2π) and *J*_m_ that is the Jones matrix of the mirror.

Considering the sensor configuration presented in Figure 2a, the complex amplitudes of the orthogonal vectors from PBS can be obtained based on *A*_ba_(0) and *J*_pc_ that is the Jones matrix of PC. The corresponding power signals after detection with OPM are defined as *S_x_* and *S_y_*. Applying the quadrature processing [21], a sensor output *S* is obtained as (*S_x_* − *S_y_*)/(*S_x_* + *S_y_*).

It is known that the expected output is obtained in the case that the linear birefringence is completely suppressed by the induced circular birefringence. In the expected case, the equivalent Jones matrix of the CSF in each section can be given by

(12) $$J_{f}=\left[\begin{array}{cc} \cos\left(F\right) & -\sin\left(F\right)\\ \sin\left(F\right) & \cos\left(F\right) \end{array}\right],\;J_{b}=\left[\begin{array}{cc} \cos\left(F\right) & \sin\left(F\right)\\ -\sin\left(F\right) & \cos\left(F\right) \end{array}\right],$$

where *F* is the Faraday rotation angle, *F* = *VHl*. The total length *l* of the CSF in each section can be derived as

(13) $$l=\int_{0}^{2\pi }\rho \cdot d\varepsilon =num\cdot \sqrt{4\pi^{2}r^{2}+h^{2}},$$

Thus, in the expected case, the Jones matrix of the sensor head shown in Figure 2a can be given by

(14) $$J_{sh}=\underset{Back\;Propagation}{\underset{︸}{J_{b}\cdot J_{b}\cdot J_{b}\cdot J_{b}}}\cdot \underset{mirror}{\underset{︸}{J_{m}}}\cdot \underset{Forward\;Propagation}{\underset{︸}{J_{f}\cdot J_{f}\cdot J_{f}\cdot J_{f}}},$$

On this basis, referring to the calculation of the *S*, the expected output can be obtained, which is defined as *S*_0_. The output error of stray current sensor can be given by Equation (15), which is defined as *U*. According to the output error requirement proposed by Guangzhou metro, the *U* caused by the linear birefringence should be smaller than 0.5% in the stray current measurement.

(15) $$U=\left|\frac{S-S_{0}}{S_{0}}\right|\times 100\%,$$

## 4. Parameter Optimization of the CSF

The known parameters of our sensor head include: *a* = 30 mm; *b* = 70 mm; *n*_1_ = 400 /m; *n*_2_ = 400 /m; *r* = 2 mm. The unknown key parameters are *Len* and *num*, respectively. The length of each curve spiral *h* is equal to the *Len*/*num*. Moreover, given our processing condition, *Len* is set within the range from 300 mm to 600 mm while *num* is set within the range from 1 to 64.

**Figure 5:**
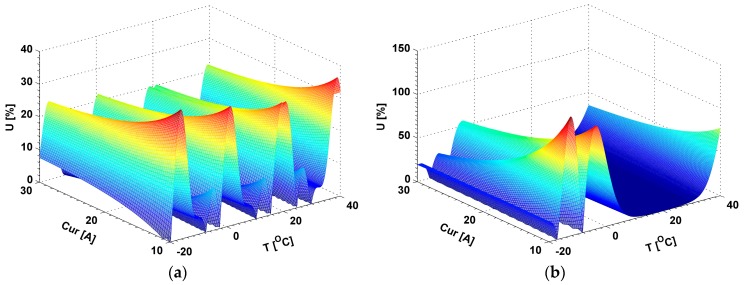
The simulation results to evaluate the effect of the *Len* & *num* on the output error U: (**a**) *Len* = 600 mm and *num* = 64; (**b**) *Len* = 300 mm and *num* = 1.

Two simulations have been conducted to evaluate the effect of these two unknown key parameters on the output error. During the simulations, the applied current (Cur) is set within the range from 10.0 A to 30.0 A based on our test results in Guangzhou metro, which is performed at 20 °C. The temperature is changed within the range from −20 °C to 40 °C. We define the total temperature-induced linear birefringence, the total bending-induced linear birefringence and the total circular birefringence of the CSF in each section as TTILB, TBILB, and TCB in this work. In the first simulation, the *Len* is set as 600 mm and the *num* is set as 64. According to Equation (13), the length of the CSF in each section is about 722 mm. According to Equations (7) and (8), the maximum TTILB is about 906 deg at −20 °C. According to Equation (6), the TBILB is about 8349 deg. Moreover, the torsion *τ* can be obtained based on Equation (2), and the TCB is about 19139 deg. This simulation result is shown in Figure 5a. The maximum U is about 39.6%. In the second simulation, the *Len* and *num* are set as 300 mm and 1, respectively. The length of the CSF in each section is about 300 mm. The TBILB, maximum TTILB, and TCB are about 0.0069 deg, 377deg, and 360 deg, respectively. The simulation result is shown in Figure 5b. The maximum U is about 136.3%. In Figure 5, it can also be found that the variation of the output error with the temperature changes in the first simulation is different from that in the second one. It indicates that the selections of these two unknown key parameters have a great influence on the output error. We believe obtaining the optimum results of these unknown parameters (*Len* & *num*) efficiently and accurately may be a challenge.

**Figure 6:**
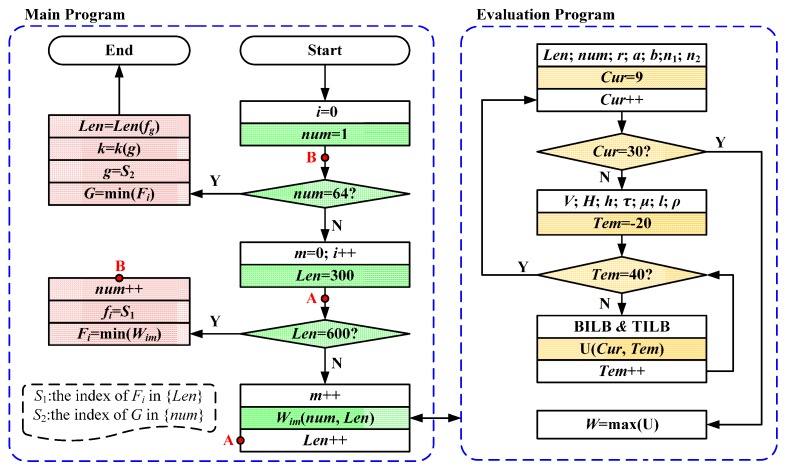
The flowchart on the optimization of *Len* and *num* in this work.

As far as we know, the optimum results are usually difficult to be obtained based on the common optimization algorithms, such as the genetic algorithm (GA) and the particle swarm optimization (PSO) algorithm. Thus, we proposed a direct search method to obtain the optimum results of the unknown parameters in this work. Compared with the GA or the PSO, our method can obtain the optimum results accurately, except for requiring more searching time. The flowchart of our method is shown in Figure 6, which includes the main program and the evaluation program. The *num* is searched within the range from 1 to 64 while the *Len* is searched within the range from 300 to 600. The searching spaces of *num* and *Len* are defined as {*num*} and {*Len*}, respectively. In the main program, the evaluation function result *W_im_* can be obtained at the *i*th *num* and the *m*th *Len*. On this basis, there are two steps to obtain the optimum results of the unknown parameters. Firstly, at the *i*th *num*, the minimum result and its index in the {*Len*} are obtained, which are defined as the *F_i_* and *S*_1_. Secondly, the minimum results of {*F_i_*} and its index in the {*num*} are obtained, which are defined as *G* and *S*_2_. Finally, the optimum results of *num* and *Len* can be found according to the indexes *S*_1_ and *S*_2_. Moreover, the evaluation program shows the physical structure of the evaluation function. At the *i*th *num* and the *m*th *Len*, the maximum output error can be calculated within the range of the stray current from 10 A to 30 A and of the temperature from −20 °C to 40 °C. In this work, we consider the maximum output as the evaluation function result at the *i*th *num* and the *m*th *Len*. It is noted that the theoretical bases of the evaluation program are Equations (1) to (15).

**Figure 7:**
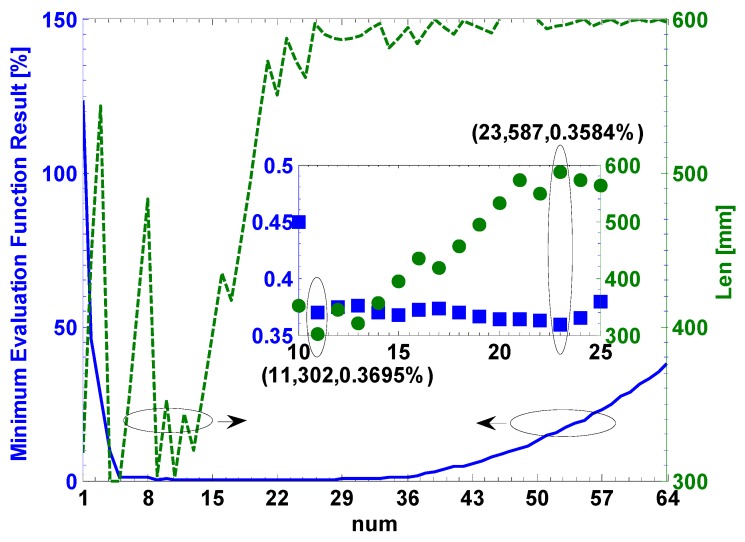
The MEFR versus the unknown parameters.

Figure 7 shows the minimum evaluation function result (MEFR) versus the unknown parameters (*num* & *Len*). The optimum results are that the *num* is 23 and the *Len* is 587 mm, where the MEFR is about 0.3584%. Moreover, the MEFRs in the range of the *num* from 10 to 25 are compared, which is shown in the inserted picture of Figure 7. It can be found that the MEFR is about 0.3695% when the *num* is 11 and the *Len* is 302 mm. Obviously, the MEFR in the latter case (about 0.3695%) can be little more than the optimum case (about 0.3584%). However, the two unknown parameters of the latter case are smaller than the optimum case, which may lead to the manufacture of the latter case being much easier. Thus, in this work, the unknown parameters *num* and *Len* are set as 11 and 302 mm, respectively.

**Figure 8:**
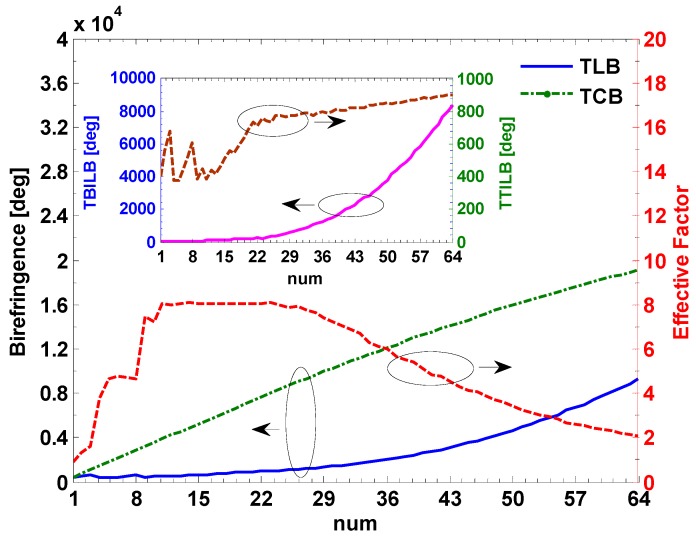
The birefringence and effective factor versus the unknown parameters.

In Figure 7, the change of the MEFR includes three cases: firstly decreases when the *num* increases from 1 to 9, then vibrates when the *num* increases from 10 to 25, finally increases when the *num* increases from 26 to 64. Figure 8 shows the birefringence and the effective factor versus the unknown parameters. The TBILB increases from 0.006 deg to 8407.1 deg while the maximum TTILB (at −20 °C) increases from 377.8 deg to 904.3 deg when the *num* increases from 1 to 64. They are shown in the insert picture of Figure 8. We defined the total linear birefringence of the CSF in each side as TLB. The maximum TLB is the sum of the TTILB and the TBILB, which increases from 379.6 deg to 9311.4 deg. Moreover, the TCB increases from 359.9 deg to 19119.2 deg. We proposed the effective factor (EF) to express the change of the MEFR, which is given by:

(16) EF=TCB/TLB

In the first case, the EF increases from 0.902 to 7.427 as the *num* increases. The MEFR decreases from 123.3% to 0.42%. In the second case, The EF vibrates within the range from 7.23 to 8.07 and the MEFR also vibrates within the range from 0.358% to 0.449%. Among them, the minimum MEFR is about 0.358% at *num* = 23 and *Len* = 302 mm when the EF is about 8.07. Finally, in the last case, the EF decreases from 7.93 to 2.05 and the MEFR increases from 0.372% to 37.96%. In this work, the MEFR is required to be smaller than 0.5%, which indicates that most of the linear birefringence should be suppressed by the circular birefringence produced by the CSF. Thus, the EF needs to be greater than 7.42.

**Figure 9:**
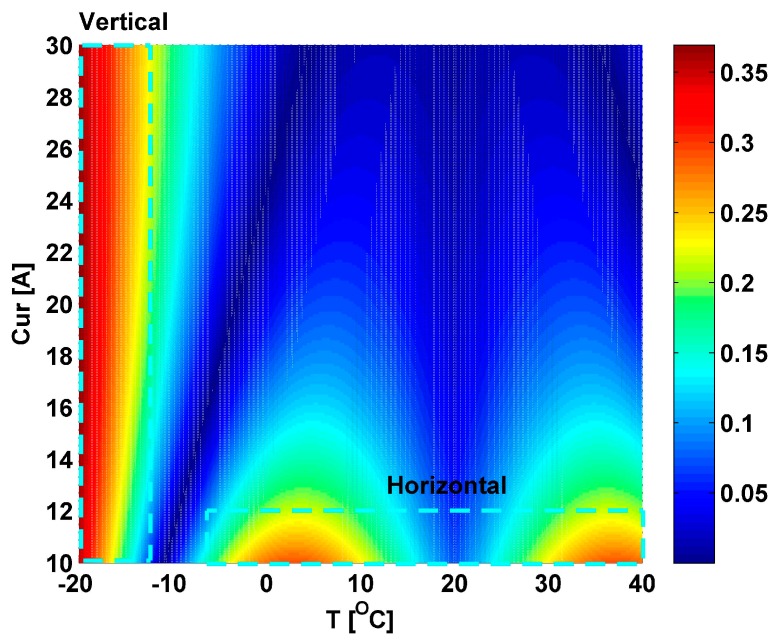
The output error distribution of our sensor using this CSF when the Cur is changed within the range from 10.0 A to 30.0 A and the *T* is changed within the range from −20 °C to 40 °C.

From discussion above, the *num* and the *Len* of the CSF in each side are set as 11 and 302 mm, respectively. The theoretical results using this CSF are calculated as follows: the length of the CSF in each section is about 309.8 mm; the TBILB, maximum TTILB, and TCB are about 92.33 deg, 388.78 deg, and 3860.2 deg, respectively. The EF using this CSF is not smaller than 8.02. Moreover, the output error distribution of our sensor using this CSF is shown in Figure 9 when the Cur is changed within the range from 10.0 A to 30.0 A and the temperature (*T*) is changed within the range from −20 °C to 40 °C. It can be found that the maximum output error is about 0.3695% as the temperature changes, which achieves the output error requirement that is not greater than 0.5%. In Figure 9, we can also find that there are two sections with relatively large output error. The vertical section is caused in the range of the *T* from −20 °C to −17 °C, where the TILB is relatively large. The horizontal section is caused in the low stray current range of about 10.0 A to 12.0 A. It is noted that the Faraday effect induced by the applied stray current can be considered as the circular birefringence [20], which is proportional to the applied stray current. Therefore, at the same temperature condition, most of the linear birefringence can be suppressed by the circular birefringence induced by the CSF, the effect of the residual linear birefringence that is not suppressed may be more significant in the low current (10.0 A to 12.0 A) than the high current (≥12.0 A), which is due to the low Faraday effect in the low current.

## 5. Verification Test

According to the discussion above, the linear birefringence and the circular birefringence work together in the CSF. It is a complex measurement problem and there are no accurate methods to measure the mutual coupling birefringence in the meter-long CSF at present. Thus, the direct measurement on the linear or circular birefringence cannot be performed in this work. The other verification method is applied, that is, the temperature experiments are conducted to verify the feasibility and effectiveness of the CSF. In the temperature experiments, the SLD and the OPM are both produced by EXFO Co. Ltd. (mode FLS-2200 and PM-1623, Quebec, QC, Canada). The 3 dB spectral width of SLD is about 56 nm. The extinction ration of LP is about 35 dB and that of PBS is about 31 dB. The coupling ratio of coupler is about 50:50. Moreover, it is noted that the detection result of a digital multimeter (Agilent Co. Ltd., model U3402A, Santa Clara, CA, USA) is used as the standard value of the experiment current. Accuracies for the DC current and voltage are 0.05% and 0.012%, respectively. The corresponding measuring ranges are smaller than 12 A and greater than 120 mV. If the experiment current is greater than 12 A, a precision shunt (type 25A/250 mV) will be required. The parameters of the CSF in each side are summarized as follows: the radius of the cylindrical silica rod is about 2 mm; the length of the cylindrical silica rod is about 302 mm; the number of the curve spirals is about 11; the CSF is the low-birefringence fiber, where the inherent linear birefringence is about 4 m/4° (Oxford Electronics Co. Ltd., model LB 1550-125, Hants, UK).

**Figure 10:**
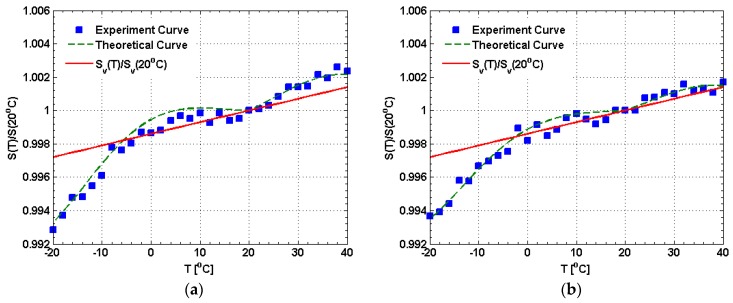

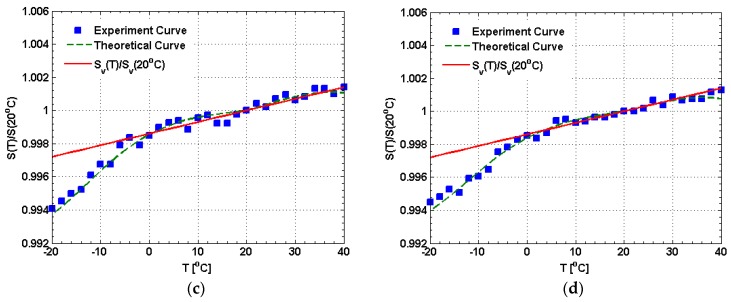
The results of the temperature experiments for different input currents: (**a**) the input current is about 15.0 A; (**b**) the input current is about 20.0 A; (**c**) the input current is about 25.0 A; (**d**) the input current is about 30.0 A.

During the following temperature experiments, the sensor output is measured as a function of temperature, for different input current. The temperature experiments are conducted by adjusting the temperature of sensor head from −20 °C to 40 °C based on a high-low temperature test chamber. The sampling is carried out every 2 °C. The temperature experiment results are shown in Figure 10, which are all normalized to the sensor output at 20 °C. For example, the normalized output at 20 °C is equal to 1. If the linear birefringence is suppressed completely by the circular birefringence produced by the CSF, the temperature dependence of the stray current sensor will arise primarily through the change in the Verdet constant with temperature. The normalized form (d*V*/d*T*)/*V*_0_ can be used to represent the temperature dependence of the Verdet constant, where *V*_0_ is the Verdet constant of the CSF at 20 °C. It is noted that the (d*V*/d*T*)/*V*_0_ is about 7 × 10^−5^/°C [22]. Thus, only considering the temperature dependence of the Verdet constant in the case that the linear birefringence is suppressed completely, the normalized sensor output as the temperature changes can be expressed by

(17) $$\frac{S_{V}\left(T\right)}{S_{V}\left(20\;\mathrm{{}^{\circ}C}\right)}=7\times 10^{-5}T+0.9986$$

Thus, the error at some temperature is the difference between the normalized output at this temperature and that at 20 °C, including the first error caused by the temperature dependence of the Verdet constant and the second error caused by the linear birefringence. Among them, the first error can be obtained based on Equation (17). Figure 10a–d are obtained at 15.0, 20.0, 25.0, and 30.0 A, respectively. It can be found that the experiment curves gradually trend to the temperature dependence curve based on Equation (17) as the input current increases, especially within the range of the temperature from 0 °C to 40 °C. It means that the effect of the linear birefringence can be eliminated more effectively as the input current increases, which is consists with the discussion of the horizontal section in Figure 9. In Figure 10a, excluding the contribution of the temperature dependence of the Verdet constant (the first error), the maximum second error is occurred at −20 °C that is about 0.43% compared with 20 °C. Similarly, in Figure 10b–d, the maximum second error is about 0.35%, 0.33%, and 0.32%, which all occurred at −20 °C. These results indicate that the effect of the most of the linear birefringence can be eliminated effectively by the circular birefringence produced by the CSF. The measurement error caused by the linear birefringence can be controlled at less than 0.43% which achieves the output error requirement (≤0.5%). It is noted that the temperature dependence of the Verdet constant (the first error) can be compensated based on the neural network or database technology, which will be our future work.

## 6. Conclusions

In this paper, an elimination method of the TILB in the stray current sensor is proposed using the CSF that can produce a large amount of circular birefringence based on geometric rotation effect. The differential equations that indicate the polarization evolution of the CSF element are derived. The output error model is built based on the Jones matrix calculus and then an accurate search method is proposed to obtain the key parameters of the CSF, including the length of the cylindrical silica rod and the number of the curve spirals. The optimized results are 302 mm and 11, respectively. Moreover, an effective factor is proposed to analyze the elimination of the TILB, which should be greater than 7.42 to achieve the output error requirement (≤0.5%). Finally, the temperature experiments are conducted to verify the feasibility of this elimination method. The effect of the temperature dependence of the Verdet constant is discussed. The experiment results indicate that the output error caused by the linear birefringence can be controlled less than 0.43% using the CSF within the range from −20 °C to 40 °C. The proposed methods can also provide reference for the elimination of the temperature-induced linear birefringence in other types of OFCSs, such as the interferometer OFCS.
